# Fellowship training: a qualitative study of scope and purpose across one department of medicine

**DOI:** 10.1186/s12909-017-1062-5

**Published:** 2017-11-21

**Authors:** Jolanta Karpinski, Rola Ajjawi, Katherine Moreau

**Affiliations:** 1 Department of Medicine, University of Ottawa, Rm 5-16, 1967 Riverside Drive, Ottawa, Ontario K1H 7W9 Canada; 2 Centre for Medical Education, University of Dundee, Dundee, UK; 3 Faculty of Education, University of Ottawa, Ottawa, Ontario Canada

**Keywords:** Fellowship training, Postgraduate medical education, Qualitative research

## Abstract

**Background:**

Fellowship training follows certification in a primary specialty or subspecialty and focusses on distinct and advanced clinical and/or academic skills. This phase of medical education is growing in prevalence, but has been an “invisible phase of postgraduate training” lacking standards for education and accreditation, as well as funding. We aimed to explore fellowship programs and examine the reasons to host and participate in fellowship training, seeking to inform the future development of fellowship education.

**Methods:**

During the 2013–14 academic year, we conducted interviews and focus groups to examine the current status of fellowship training from the perspectives of division heads, fellowship directors and current fellows at the Department of Medicine, University of Ottawa, Canada. Descriptive statistics were used to depict the prevailing status of fellowship training. A process of data reduction, data analysis and conclusions/verifications was performed to analyse the quantitative data.

**Results:**

We interviewed 16 division heads (94%), 15 fellowship directors (63%) and 8 fellows (21%). We identified three distinct types of fellowships. Individualized fellowships focus on the career goals of the trainee and/or the recruitment goals of the division. Clinical fellowships focus on the attainment of clinical expertise over and above the competencies of residency. Research fellowships focus on research productivity. Participants identified a variety of reasons to offer fellowships: improve academic productivity; improve clinical productivity; share/develop enhanced clinical expertise; recruit future faculty members/attain an academic position; enhance the reputation of the division/department/trainee; and enhance the scholarly environment.

**Conclusions:**

Fellowships serve a variety of purposes which benefit both individual trainees as well as the academic enterprise. Fellowships can be categorized within a distinct taxonomy: individualized; clinical; and research. Each type of fellowship may serve a variety of purposes, and each may need distinct support and resources. Further research is needed to catalogue the operational requirements for hosting and undertaking fellowship training, and establish recommendations for educational and administrative policy and processes in this new phase of postgraduate education.

## Background

In Canada, completion of undergraduate medical school education confers a medical degree and leads to graduate training in family medicine (2 years) or one of 65 medical, surgical or diagnostic specialties or subspecialties (4–6 years depending on the discipline). As an example, certification in the subspecialty of Nephrology requires completion of medical school, 3 years of Internal Medicine specialty residency and 2 years of Nephrology subspecialty residency, with success in the certification exam of each discipline. This residency training occurs under the auspices of the College of Family Physician of Canada (for family medicine) or the Royal College of Physicians and Surgeons of Canada (for specialty and subspecialty training) in partnership with Canada’s 17 Faculties of Medicine. These governing bodies have established goals, objectives and requirements for residency training, rigorous assessment processes with national standardized examinations as well as robust accreditation standards and processes to ensure a high quality educational standard and outcome.

Increasingly, graduates of specialty and subspecialty residency programs, having achieved Royal College of Physicians and Surgeons of Canada certification and the prerequisites for independent practice, are choosing to pursue additional non-accredited training to attain distinct and advanced clinical and/or academic skills. This phase of medical education, confusingly called “fellowship training”, has been an “invisible phase of postgraduate training” [1] lacking standards for education and accreditation, as well as lacking funding. However, the number of fellowship programs is increasing [2, 3], as are the number and proportion of residents pursuing fellowship training [4–8]. This has been well documented in the surgical disciplines; surveys indicate that 60–85% of Canadian residency graduates from general surgery [6], radiology [7] and urology [8] intend to pursue a fellowship, with similar findings from studies in the UK and US [9, 10].

Surveys (trainees) and opinion pieces (faculty) have offered reasons for this growth in post-residency education. Trainees’ reasons for pursuing fellowship training include attaining clinical competence, increasing confidence and attaining specialized skills [10] as well as pursuit of an academic career, the acquisition of marketable skills and the achievement of specific career goals [6–8, 11]. From the faculty perspective, fellowship programs have been proffered as a solution to the problem of lack of after-hours clinical coverage [12] and have been noted to provide academic and clinical benefits with better teaching for more junior learners, facilitation of research productivity and improvement in volume and quality of clinical services [5, 12, 13]. These studies are based on self-reports and are limited by their focus on a single program, or a single area of interest (e.g. rhinology). There is a lack of literature identifying the purpose and benefits of fellowship programs in medical disciplines, and viewed from the perspective of an academic institution such as a department or faculty of medicine.

The Department of Medicine (DoM) at the University of Ottawa administers residency training in 5 primary specialties (Internal Medicine, Dermatology, Neurology, Nuclear Medicine, and Physical Medicine and Rehabilitation) as well as 13 subspecialties (e.g. Cardiology, Nephrology, Rheumatology). Individual divisions have been attracting subspecialty graduates and developing fellowship programs without oversight or a central plan from the Department, Faculty of Medicine or other education bodies. As fellowship programs proliferate, there is a need to understand this emerging phase of medical education as a first step in developing support and oversight. This paper explores fellowship training across all divisions in one department of medicine to describe the breadth of fellowship activity, and to identify the purpose of fellowship programs in an academic institution.

## Methods

This study represents one component of a larger mixed methods project that aimed to describe and analyse the current status as well as the educational, administrative and organizational needs of fellowship training at the DoM, University of Ottawa, Canada. We used semi-structured interviews and focus groups to gather information about the scope and purpose of current fellowship training activities. This study received ethics approval from the Ottawa Health Science Network Research Ethics Board. Participants provided written consent.

### Study populations

We identified three groups of individuals as potential informants to the study: division heads, fellowship directors and current fellowship trainees (fellows). A division head is a faculty member with the departmental responsibility for the clinical and academic administration of a clinical division. A fellowship director is a faculty member responsible for the administration of a fellowship training program. In order to encourage free and open discussion, individual interviews were chosen for the populations perceived as professional colleagues (division head and fellowship directors) and a focus group approach was selected for the fellows.

### Participant recruitment

We purposefully pursued inclusion of relevant stakeholders in order to gather data from all viewpoints of the fellowship issue. All division heads, fellowship directors and current fellows within the DoM were eligible. The sampling strategy used both initial and theoretical sampling; recruitment stopped when saturation of information was obtained.

We obtained the list of division heads from the DoM website. The research ethics board required that contact with the fellowship director and fellow study populations occur only through the division head. We contacted division heads directly by email, and sent a description of the study, the consent form and an invitation to participate. Subsequently, we used snowball sampling to identify potential participants for the fellowship director interviews. We asked division heads, via email, to forward invitations, study descriptions and consent forms to all fellowship directors in their divisions; invitations were sent twice at 2 week intervals. Similarly, we requested that division heads distribute the invitation to participate to current fellows, including a description of the study and consent form.

The primary author conducted the interviews and focus groups. This individual is a faculty member within the DoM and a clinician educator who has experience conducting qualitative interviews and focus group research. A trained qualitative research associate attended the sessions with fellows to observe and record non-verbal gestures and key points. Informed consent was obtained at the time of meeting, and the interviews and focus group sessions were audiotaped with permission. Field notes were made following each interview and focus group; these summarized key points, identified additional probes for subsequent interviews, documented the research process and tracked the development of insights and themes, which contributed to the trustworthiness of the analysis.

### Instrument development

We based the one-on-one and focus group interview guides on the major research question that guided this phase of the study (i.e. research question: what is the breadth and purpose of fellowship programs in the DoM) and used a series of semi-structured questions, with prompts, to guide the interview/focus group. The questions in the interviews and focus groups focused on a description of the existing fellowship programs; for example, tell me about your fellowship program; how is your fellowship program preparing you for the next stage in your career. Table 1 provides a list of the major questions used in the interviews and focus groups as well as examples of probing questions. We pilot tested the interview guide with two individuals who had knowledge of the topic but did not participate in the study: one graduated fellow and one program director. Information from these pilot interviews was used to improve the prompting questions and wording of the interview guide. Conversely, we had two qualitative medical education researchers, who are versed in the topic area, review the focus group guide to ensure that we had worded the questions appropriately and that we were not missing any pertinent questions. We also reviewed the focus group question guide after the first focus group, refining the probing questions to ensure that necessary topics were fully explored. Based on this review, we did not need to make any substantive changes.

**Table 1:** Interview and focus group questions

Interview Questions
1. Describe the fellowship(s) offered by your division
Example probes:
a. What additional skills does the trainee obtain during their fellowship? (Clinical, education, research?) How long is it?
b. How long has it been in place? Why was it started?
c. What is working well in your fellowship program?
2. What are the benefits of offering a fellowship program?
3. What challenges does your fellowship program face?
Example probes
Administrative challenges? Educational/supervision challenges?
4. What would improve your ability to support a successful fellowship program?
Focus Group Questions
1. Tell me about your fellowship program
Example probes
What are you learning in your program?
What is working well in your program?
2. How is your fellowship program preparing you for the next stage in your career?
Example probes:
a. What are you expecting to do after this fellowship?
b. Has/will the fellowship prepare you well for that next step?
3. What would you improve in your fellowship program?

*Note.* Probes used for each question varied depending on the flow of and information provided by the participants

### Data analysis

The audio recordings were transcribed verbatim by a professional transcriptionist. The primary author compared the transcripts to the audio-recording to verify the accuracy of transcription. We used NVIVO 10 (QSR International), a computer-assisted qualitative data analysis software program, to store, organize and manage the qualitative data. Simple counts were used to depict the prevailing status of fellowship training in the DoM (e.g. number of fellows per year).

To analyze the interview transcripts, we applied a cyclical and iterative analytic process using three concurrent activities—data reduction, data analysis, and conclusions/verifications. Beginning with the research question [14], a mixture of deductive and inductive coding allowed for codes that were not identified a priori to emerge from the data [15]. This process ensured that key ideas were not missed and data was not forced to fit into pre-existing codes, while ensuring the pragmatic design would respond to the research question.

The primary author analyzed all the transcripts. Throughout the process of data analysis, the primary author used constant comparison to compare the use and content of codes within each transcript, and subsequently between different study populations [16, 17]. Early in the study, and to enhance the trustworthiness of the analysis, the primary author and the above-mentioned research associate coded four different transcripts independently (i.e., two interview transcripts and two focus group transcripts). Following their analysis, they met to review their coding and discuss any inconsistencies. After discussion, they revised their coding as needed.

## Results

### Demographics

We completed interviews with 16 (of 17, 94%, 14 male, 2 female) division heads, four of whom also functioned as the fellowship director for their division. We identified another twenty fellowship directors through the division head interviews and 11 (10 male, 1 female) representing programs in eight divisions were interviewed (15 of 24 fellowship directors, 63%). Eleven fellows registered for, and 8 (6 male, 2 female) participated in one of two focus group sessions (5 in a first session, 3 in a second; 21% of current fellows). Participants had completed a range of 2–20 months of fellowship training, included 5 Canadian and 3 foreign trainees, and represented fellowship programs in 6 divisions.

### Range of fellowship training activity

There was a broad range of engagement in fellowship training amongst the 17 divisions (see Table 2), with a total of 26 distinct fellowship programs (e.g. Electrophysiology, Laser dermatology, Thrombosis, Advanced GI therapeutics). Fifteen divisions had fellows in training within the previous 3 years. Thirteen were currently active, with the number of trainees ranging from 1 to 12 per division. Seven divisions offered fellowships in more than one area.

**Table 2:** Department of Medicine fellowship programs

Division	Number of fellows/year	Duration (yrs)	Focus
Cardiology	12–15	2	Advanced clinical skills in 5 distinct areas
Critical Care Medicine	2	2	Individualized
Dermatology	1	1	Advanced clinical skills in one area
Endocrinology and Metabolism	0–1	2	Individualized
Gastroenterology	2	1	Advanced clinical skills in two distinct areas
General Medicine	1–2	1	Individualized
Hematology	5	2	Two areas of focus: Advanced clinical skills in two distinct areas Research
Infectious Diseases	0–1	1–2	Individualized
Medical Oncology	4–6	2	Research
Nephrology	5–6	1–2	Advanced clinical skills in two distinct areas
Neurology	4–5	2	Advanced clinical skills in three distinct areas
Palliative Care	0–1	Variable	Individualized
Physical Medicine and Rehabilitation	0–2	1	Advanced clinical skills in two distinct areas
Respirology	1	1	Advanced clinical skills in two distinct areas
Rheumatology	0–1	2	Individualized

### Focus of fellowship programs

Through the analysis of themes elucidated in the interviews and focus groups, the focus of fellowship training was categorized into one of three distinct types: individualized, research or clinical. Only one division offered fellowship programs that fell into more than one category.

#### Individualized fellowships

Individualized fellowships were arranged in response to the individual career goals of the trainee and/or the recruitment goals of the division. As a participant described,

*We do it very individually, candidate focused, so it really depends on the career path of the individual who wants to do it. So we try to accommodate those different career paths (Division head)*

The defining feature of this category was the focus on individual interests and/or specific divisional need. These fellowships were often associated with enrollment in a graduate degree program (Masters). The trainees’ clinical activities were associated both in content and in degree of autonomy with their current credentials; that is, the clinical work of these trainees was linked to the discipline of their previous training and usually not related to the acquisition of new clinical skills. The duration of this training was typically linked to that of the degree program, usually 2 years.

#### Research fellowships

Fellowship programs focused on research were defined by their sustained programmatic approach to research. The focus was on research productivity, as defined by dissemination. As a participant described,

*We try to set the bar for our Fellows, five to seven publications per year that they’re here*

*(Fellowship director)*

There was a strong research program in the division, and excellent research mentors. These fellowships could also be linked to the attainment or refinement of clinical skills, but the clinical aspects were not the main focus of activity.

#### Clinical fellowships

The majority of fellowship programs focused on the attainment of clinical expertise over and above the competencies of specialty or subspecialty residency, usually in a distinct clinical area.

*We have a one year stream where Fellows are dedicated to clinical work only and we have a series of objectives that they should be obtaining by the end of the one year program (Fellowship director)*

The duration was generally 1 year, but some extended to 2 years. These trainees were supervised in the clinical environment, as appropriate to the development of new skills, but given the advanced standing of this group of learners, the supervision could be minimal. Research was not a major feature of most of these programs, and, when present, was tightly linked to the clinical focus. These programs were usually focused on a specific disease entity (e.g. sleep medicine, stroke, heart failure) or treatment modality (e.g. interventional cardiology, renal transplantation).

### Purpose of fellowship programs

Through the analysis of themes elucidated in the interviews and focus group, it was identified that faculty members and trainees offered similar reasons for deciding to pursue fellowships. All three study populations identified the purpose of fellowship to be academic productivity, clinical expertise, recruitment and reputation. In addition, division heads and fellowship directors identified the benefits of fellowship programs on clinical productivity and the scholarly environment.

#### Academic productivity

Fellowship training was perceived to enhance scholarly output directly through the academic activities that fellows pursued in terms of research and publications. The presence of fellows performing clinical activities was also seen as freeing up the time of faculty members for their own academic work and therefore indirectly increasing scholarly output.

*The fellows, in providing a clinical service, protect the time of scientists so that’s one mechanism of reducing clinical load. The fellows write papers, the fellows write grants; it increases the productivity of our staff (Division head)*

*It’s our job to churn our papers. It’s our job to publish. It’s our job to go to meetings or present posters. It’s our job to write protocols. (Fellow)*

#### Clinical expertise

A desire to share and/or develop enhanced clinical expertise was the main motivation for many of the clinical fellowship programs. Division heads and fellowship directors expressed the importance of being able to train individuals beyond the skills learnt in specialty and subspecialty programs, identifying unique patient populations in need of these skills and/or new or emerging advances and treatments in their field. For trainees, additional expertise led to added confidence in the clinical realm and provided new opportunities for future clinical practice.

*There’s a huge clinical need for people with this training so to be able to provide that training is important (Fellowship director)*

*Having the extra expertise in “XX” allows me to go to an underdeveloped area of the country afterward and be able to have a bit more authority in being able to present myself as a specialist in that particular topic (Fellow)*

#### Recruitment

Recruitment to academic positions was identified as an important aspect of fellowship programs by both faculty members and trainees. This was expressed for all three categories of fellowship programs. Individualized programs allowed division heads to identify areas of need within the division, and recruit and train a fellow to meet that need, whereas clinical and research fellowships gave the division heads an opportunity to observe a potential recruit’s performance during training. Fellows identified the completion of fellowship training as a perceived “must” in order to attain an academic position.

*They’re also our farm team. So we get to try them out for two years. Is this person going to be a star or not? And hire them at the end of the day (Division head)*

*I got talked into doing it by a supervisor who made the case that it would help to open doors career wise (Fellow)*

#### Reputation

Directors and division heads viewed the fellowships as a means to spread their influence by providing a beneficial training experience to individuals who would establish a practice in other regions or countries. Trainees perceived that the fellowship would enhance their own reputation via the means of additional training/scholarship.

*Certainly it helps with promoting Ottawa as a place with expertise if you have Fellowship programs and Fellows coming to train, they go back to wherever they’re from and they’re ambassadors (Division head)*

*We have people go all over the world. India, Mauritius, Anstralia, U.K., wherever, their training is going to reflect on excellent training that they received here. (Fellowship director)*

#### Clinical productivity

Informants described fellowship programs as a means to improve and/or increase the clinical services provided, that is to improve clinical productivity. In this instance, fellows were perceived as directly adding to the manpower of the division or as a necessary element in order to allow an increase in divisional services.

*We, like many (other places) in Ontario, have problems with wait times, [scheduling] new patients to be seen. The more fellows we have then the better a job we can do with that to get the patients seen (Division head)*

*So as our clinical program expanded, part of that plan was that we would have Fellow support or Fellow manpower (Fellowship director)*

#### Scholarly environment

Directors and division heads described an enhancement in the scholarly environment of the division as a result of the presence of this group of advanced learners. They identified improvements in both the formal and informal academic curriculum as well as benefits in the interactions with other learners in the system.

*There’s kind of a milieu element. So, basically, by having the fellows it creates this academic environment that before fellows we never had. …you have to create an atmosphere of collegiality, collaboration, academic advancement, interaction and have regular rounds and regular research meetings and this type of thing to help keep the ball rolling in the academic world. (Division head).*

## Discussion

In this report, we describe the full range of fellowship programs across one department of medicine. Whereas previous reports have focused primarily on single programs and the perspectives of trainees [2, 3, 7, 11, 13, 18–28], the new findings in this report result from its broad focus across multiple types of fellowship programs and the triangulation of views from learners, teachers, educators and administrators. We were able to categorize fellowship programs into three distinct types: individualised, research, and clinical. This taxonomy, and in particular the description of individualized fellowships, is a novel addition to the literature on post-residency training. These categories may be useful when considering the differing resources required to support fellowship programs, as well as the various desired outcomes of training.

In examining the focus and purpose of fellowship training, this study reproduces and expands upon previous reports. The fellows’ direct contributions to academic and clinical productivity have been described by others [12], but we also identify the indirect benefit on academic productivity that fellows enable by “freeing up the time” of their supervisors to engage in scholarly activities. Previous authors [13] have discussed the benefits of fellowship on the scholarly environment, but our findings expand on that concept to include improvements in the formal and informal academic curriculum as well as the teaching of more junior learners. The role of fellowships in recruitment to academic positions was described by all stakeholders and across all types of fellowship programs; this purpose of fellowship training has not previously been reported. Finally, the reputational benefits of completing fellowship training and hosting a fellowship program are also a unique contribution to the literature.

Studies reporting outcomes of fellowship training support the positive perceptions of the benefit of fellowship on an individual’s clinical expertise and academic productivity. In their first years of practice, graduates of fellowship programs have been reported to provide higher quality care, performing better than their peers on measurements of clinical performance: reduced false-positive rates on screening mammograms [29]; lower rates of positive margins in cancer surgery [30]; and, better outcomes in a variety of functional and quality of life scores after total knee arthroplasty [31]. In retrospective studies, self-reported academic achievement is better in graduates of research fellowships including more time doing research, greater career satisfaction than their peers [32] and greater likelihood of having submitted a grant proposal, received grant funding, published, achieved senior academic rank [33], and taken/obtained an academic job [18].

Strengths of this study arise from our use of qualitative design and methods, which provide rich descriptions of fellowship training and comprehensive viewpoints from all stakeholder groups. This study is novel in reporting across the breadth of an entire department of medicine, with varying degrees of engagement in fellowship training by different divisions, and a wide range of the category and focus of fellowship programs. Previous publications on the topic of fellowship training have been limited by their emphasis on a single training program [19] or on multiple programs with a similar focus of training (e.g. interventional pulmonology in North America) [3, 20, 22, 23, 25–28].

Limitations of the study include the primary investigator’s status as a member of the Department; we cannot exclude the possibility that participants filtered their comments during the interviews or focus groups because of that involvement. One author conducted all the interviews and focus groups, and it is possible that there was an implicit bias within some of the questions and probes used that may have influenced the interviewees to respond in a specific way in the interviews and focus groups. The breadth and number of focus group (fellow) participants is another potential limitation. However, the fellows, although small in number, represented a range of programs, a range of duration of training, and male and female gender. Moreover, the analysis of the second focus group did not identify any new coding categories. The number of months of fellowship experience was not specifically considered in the analysis of the data, as it was assumed that the purpose of undergoing fellowship was part of a decision made prior to its initiation; we cannot exclude that participation in fellowship training may reveal other perspectives on the purpose of fellowship of which the fellows were unaware at the time of the focus group discussion. Lastly, this study focused on one medical department and the transferability of the findings to other fields within medicine and surgery, and other university contexts may be questioned. This limitation may be mitigated by the broad range of fellowships under study, which included those focused on technical skills.

Opportunities for further study and exploration of fellowship training include an examination of the perspectives of other stakeholders in medical education, as well as further analysis of the educational, administrative and organizational needs to host and develop these programs. Surveys and case log studies [4, 34, 35] provide conflicting reports on the effect of the presence of fellowship programs on residents’ procedural opportunities. The perspectives of residents in primary specialties and subspecialties as well as their program directors could be further examined and compared to the fellowship directors’ perspective on the benefits of fellowship on the academic environment. Published descriptions of fellowship programs have primarily used surveys of program faculty or trainees as the sources of information. There is minimal literature describing the educational aspects of fellowship training; articles provide information on clinical and procedural volumes as a description of the resources available for clinical training, but there is little information regarding objectives, curriculum or assessment methods. Educational material, and administrative policies and procedures may be a source of information on the status of fellowship training. For example, funding, specifically source of salary funding for the trainee, is frequently stated as a common challenge and even barrier to fellowship training [3, 13, 18, 19, 28, 36]. A comprehensive review [21] highlighted the limited status and quality of publications in general, and in Canada in particular, raising questions about how to optimize training and assessment, how to ensure a proper learning environment, how to assure integration with resident training and how to address the needs of specific populations of fellows, such as those from other countries. Thus, the needs and challenges of fellowship training have yet to be fully identified, and offer opportunities for further investigation.

This exploratory study contributes classification of the focus and purpose of fellowship training to the literature on postgraduate medical education. A clear categorization and explicit statement of purpose of fellowship training facilitates further research as well as further development of this stage of professional development. As an example, an informal review of university websites has shown that, to date, faculties of medicine in Canada have generally folded the oversight of fellowship training under the auspices of the residency medical education office. However, the types of training offered in fellowships, the goals of the fellows and the hosting department, and the administrative challenges (e.g. funding) may warrant distinct infrastructure and support.

## Conclusion

This paper provides a comprehensive description of fellowship training activity at one department of medicine. It offers a novel taxonomy for describing three categories of fellowship training programs, each with its own distinct focus. Fellowship programs serve multiple purposes, each of value to the department, the faculty members and the trainees. Further research is needed to determine how best to support this phase of medical education and ensure it meets its goals, and the high standards and expectations of society.

## Abbreviations

DoM: Department of medicine
